# Ultrasonic Obstacle Avoidance and Full-Speed-Range Hybrid Control for Intelligent Garages

**DOI:** 10.3390/s24175694

**Published:** 2024-09-01

**Authors:** Lijie Wang, Xianwen Zhu, Ziyi Li, Shuchao Li

**Affiliations:** 1 School of Measurement-Control Technology and Communications Engineering, Harbin University of Science and Technology, Harbin 150080, China; wlj@hrbust.edu.cn (L.W.); 2220600066@stu.hrbust.edu.cn (Z.L.); 2420600019@stu.hrbust.edu.cn (S.L.); 2 The Higher Educational Key Laboratory for Measuring & Control Technology and Instrumentation of Heilongjiang Province, Harbin University of Science and Technology, Harbin 150080, China

**Keywords:** intelligent garage, Kalman filter, brushless DC motor, vector control, sliding mode observer

## Abstract

In the current study, which focuses on the operational safety problem in intelligent three-dimensional garages, an obstacle avoidance measurement and control scheme for the AGV parking robot is proposed. Under the premise of high-precision distance detection using Kalman filtering, a mathematical model of a brushless DC (BLDC) motor with full-speed range hybrid control is established. MATLAB/Simulink (R2022a) is used to build the control model, which has dual closed-loop vector-controlled motors in the low- to medium-speed range, with photoelectric encoders for speed feedback. The simulation results show that, at lower to medium speeds, the maximum overshoot of the output response curve is 1.5%, and the response time is 0.01 s. However, at higher speeds, there is significant jitter in the speed output waveform. Therefore, the speed feedback is switched to a sliding mode observer (SMO) instead of the original speed sensor at high speeds. Experiments show that, based on the SMO, the problem of speed waveform jitter at high motor speeds can be significantly improved, and the BLDC motor system has strong robustness. The above shows that the motor speed under the full-speed range hybrid control system can meet the AGV control and safety requirements.

## 1. Introduction

With the continuous improvements in citizens’ quality of life, the number of car owners in China is also rising. Taking the capital city of Beijing as an example, compared to the same period last year, the number of motor vehicles in the city has increased by 462,000, and it has maintained a continuous growth trend. On the other hand, due to the rigid constraints of land resources and the lag in parking space construction standards, the supply of parking spaces in many cities in China is seriously insufficient. 

The state has introduced a series of policies to solve the parking problem in a timely manner. However, the results are still slightly insufficient compared with the parking difficulties faced in our country. With the development of computer and automation technology, the intelligent garage systems are gradually emerging. As the key to the achievement of intelligent management of a three-dimensional garage, the measurement and control mode of AGV parking robots directly affects the efficiency, reliability, and safety of vehicle access. This has become a research hotspot in the field. According to public data released by various companies in the market, the use of AGVs can increase the utilization rate of a parking area by at least 40%, so the development of parking robots is very consistent with the national expectations for the construction of smart cities, and there are broad application prospects [1].

The purpose of this paper is to explore the obstacle avoidance system of AGVs. Under the condition of achieving the required accuracy for ultrasonic distance measurement of obstacles, speed control methods for brushless DC (BLDC) motors are studied. That is, during the movement of the AGV along a given trajectory, the motor control system decelerates and stops accordingly when encountering obstacles, and the local path optimization system (A* algorithm) intervenes to re-plan the trajectory or sound an alarm and stop the movement of the AGV [2]. This system is based on accurate control of the speed of the BLDC motor; after the BLDC motor converts electrical energy into mechanical energy, the AGV starts to run. The dual closed-loop control method for speed and torque in AGV driving is the research object of this paper. In order to meet the actual operational requirements of the AGV, under the premise of using a vector control algorithm to drive the motor, a photoelectric encoder is used to detect the speed in the low- to medium-speed range and a sliding mode observer is used in the high-speed stage, so as to meet the speed requirements in the full speed range. Based on the precise acquisition of the BLDC motor’s speed, the accurate displacement information of the vehicle is obtained, improving the accuracy of the vehicle’s stopping position and ensuring the safety of the AGV.

At present, the motor models of intelligent garage drive systems are different. This article selects from the following aspects.

1. Cost efficiency: in terms of cost, the price of the excitation synchronous motor and servo motor is higher, so a BLDC motor is selected.
2. Dual closed-loop control: in the case of a dual closed-loop (current outer loop and current inner loop control) control method, a BLDC motor can achieve excellent speed and torque response curves, avoiding the complex three-closed-loop control method [3].
3. Algorithm complexity: the algorithm complexity of the SMO is lower than that of a Kalman filter algorithm [4] and neural network control [5].
4. Robustness and Simplicity: it is more robust than the Luenberger observer [6], and the BLDC motor debugging process is simple.
5. Faster Response and Reduced Chattering: the BLDC motors have faster response speeds and lower chattering than fuzzy control [7] and PI control.

After careful consideration [8], a BLDC motor was selected as the drive system in this paper.

## 2. AGV Ranging System

### 2.1. Ranging with Ultrasound

**Figure 1:**
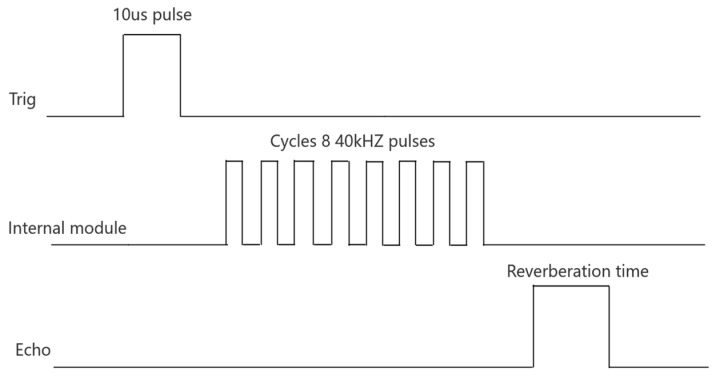
Working sequence diagram of HC-SR04.

An HC-SR04 ultrasonic sensor is selected for ranging; the ultrasonic ranging method adopts the transceiver separation method, and the basic principle of ultrasonic ranging is the time difference ranging method [9]. As shown in Figure 1, the trig pin sends a pulse to indicate that the sensor is starting to work, and the wait time for the echo pin to outputs a high-level signal (the reverberation time in Figure 1) from the HC-SR04 sensor is the time difference *t*. The microcontroller captures this time difference, and the distance to the obstacle is calculated based on the speed of sound. *S* is the distance from the ultrasonic transmitter to the obstacle, and the distance calculation formula is

(1) $$S=v\cdot \frac{t}{2}$$

where *v* is the propagation velocity of ultrasonic waves in air.

Since the speed of sound-wave propagation in air is related to temperature, temperature compensation is required under high accuracy requirements. After adding temperature compensation, the relationship between the speed of sound-wave propagation in air and temperature [9] is as follows:

(2) v=331.4+0.607T

where *T* is the ambient temperature at the time of measurement.

### 2.2. Kalman Filter Algorithm

The Kalman filter algorithm [10] first uses the linear system state equation, then, through the system input and output observation data, finally carries on the optimal estimation of the system state. Kalman filtering is widely used in various fields, especially in distance detection, and its ability to correct data is particularly significant [11]. For research on indoor ultrasonic positioning technology, the modal optimization Kalman filter algorithm is used. The algorithm optimizes the distance measured by the ultrasonic probe [12], so that the correction value is very close to the actual value, and after 50 recursions, the error is controlled within the range of ±1 cm. Therefore, the feasibility of the Kalman filter algorithm can be verified.

When laser radar ranging is applied in the fields of autonomous driving and aerospace, the ability of Kalman filter technology to correct data is particularly significant. For example, in the real-time tracking problem of missile trajectories, firstly, the accurate distance and velocity data provided by radar are used. Then, the interactive multi-model Kalman filter algorithm [13] is used to optimize the battle. Finally, through simulation experiments, the actual trajectory of the missile basically coincides with the filtered trajectory. Therefore, under the premise of considering the applicability of the three-dimensional garage, the Kalman filter algorithm is added to the measurement process to reduce the measurement error.

Kalman filter is a recursive estimation algorithm. Under the premise of minimum mean square error as the best criterion for estimation, the optimal estimation is sought over time. The basic idea is that on the basis of constructing the state space model of output and noise, the estimated value and the measured value are combined. The predicted value-measured and value-corrected value is continuously repeated, and the random error and noise of the system are eliminated according to the measured value of the system.

The output ultrasonic observation model of the ultrasonic sensor is

(3) $$\begin{array}{c} \left[\begin{array}{c} x_{1.k}\\ x_{2.k} \end{array}\right]=\left[\begin{array}{c} 1\;\;1\\ 0\;\;1 \end{array}\right]\left[\begin{array}{c} x_{1.k-1}\\ x_{2.k-1} \end{array}\right]+\left[\begin{array}{c} w_{1.k-1}\\ w_{2.k-1} \end{array}\right]\;\\ \left[\begin{array}{c} z_{1.k}\\ z_{2.k} \end{array}\right]=\left[\begin{array}{c} 1\;\;0\\ 0\;\;1 \end{array}\right]\left[\begin{array}{c} x_{1.k}\\ x_{2.k} \end{array}\right]+\left[\begin{array}{c} v_{1.k-1}\\ v_{2.k-1} \end{array}\right]\;\;\;\;\;\; \end{array}$$

where *x*_1*.k*_ *x*_2*.k*_ are the ultrasonic arrival distance and the acoustic wave propagation velocity under the prediction model, respectively; *z*_1*.k*_ *z*_2*.k*_ are the arrival distance and velocity of acoustic wave in the actual propagation model of ultrasonic wave; *w*_1.*k*−1_ *w*_2*.k*−1_ is the measurement process noise; *v*_1.*k*−1_ *v*_2*.k*−1_ is the measurement noise.

The noise *w*_1*.k*−1_ *w*_2*.k*−1_ in the measurement process is affected by the temperature and humidity and the flatness of the measured object surface. The sampling rate of the single-chip microcomputer mainly determines the measurement noise *v*_1.*k*−1_ *v*_2__.*k*__−1_. In the calculation process, the two kinds of noise can be processed according to the normal distribution [14], and the variances are set to *Q* and *R*, respectively.

### 2.3. Preprocessing Flow of Kalman Filtering

Kalman filtering is a linear system state equation. The Kalman filtering process of the output of the ultrasonic sensor is as follows [11]:

1. The optimal estimate of the previous moment is used as input for the prediction parameter of the current distance;
2. The variance of the prediction is the sum of the variance of the optimal estimate at the previous moment and the process variance. According to the influence of sensor error, car running resistance, temperature, and atmospheric pressure, this paper takes the process variance *Q* = 0.01;
3. Considering the velocity as constant, the system can be transformed into a one-dimensional Kalman filter to calculate the Kalman gain;
4. According to the measured value at the current moment, the prediction at the previous moment is corrected to obtain the optimal estimation after correction, and the variance of the final estimated value is calculated;
5. As the sampling process progresses, the process of (2)~(4) is continuously repeated.

The measurement variance reflects the measurement accuracy of the sensor. According to the sensor parameter data, *v* has been processed according to the normal distribution [12], so the *R*-value is the variance of *v*. The variance of *v* is calculated to be 0.096 by the initial measurement result, so *R* takes 0.1. The estimated variance of the initial value depends on the accuracy of the initial value setting. In this paper, *p* = 1.0 is taken.

**Figure 2:**
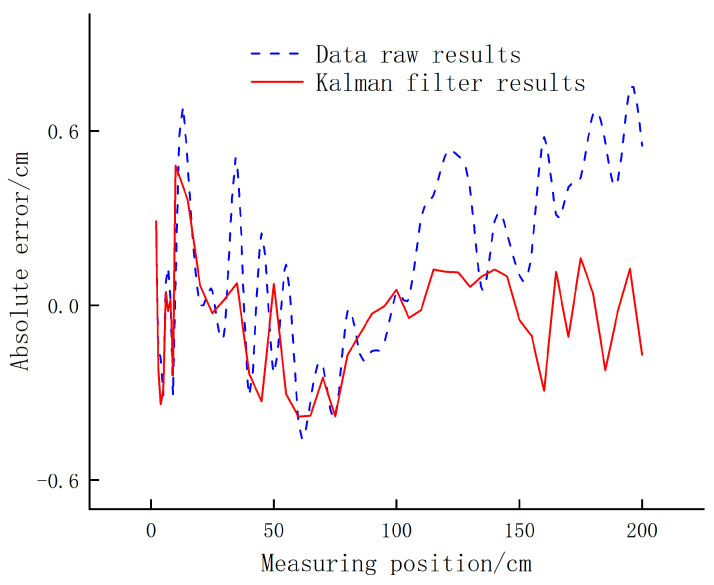
Effect of data filtering.

A horizontal obstacle is placed in the measurement range (2–450 cm) of the sensor HC-SR04, and then the ranging experiment is carried out. As shown in Figure 2, it is the absolute error of the unpreprocessed data and the Kalman filter data with the actual value, respectively, indicating that the average ranging error is reduced by 32% under the filtering effect.

Table 1 shows the error comparison after filtering under the PauTa criterion, indicating that the random interference is effectively suppressed, which meets the accuracy requirements of AGV ranging.

According to the test and data processing analysis, the results show that the average error of the measurement results after Kalman filtering is less than 0.17 cm, which is 39% lower than 0.28 cm before filtering, and the fluctuation of the sampling results after filtering is minor.

## 3. The Motor Control Algorithm

### 3.1. Establishment of Motor Model

According to the current mainstream AGV design companies, such as Hikvision, KUKA, Dematic, etc., the total driving force [15] required for AGV driving *Fz* is 1562 N. The parameters required for designing AGV are as follows: the average driving speed of AGV *Vx* is 0.58 m/s, the diameter of AGV driving wheel *D* is 18 cm, the number of driving motors *n* is 10, the reduction ratio of motor reducer *i* is 20, and the transmission efficiency of the motor output shaft from the reducer to the driving wheel is 0.73. According to these parameters, the calculation formulas of the rated power *P*, the rated torque *T*, and the rated speed *N* of the motor are Equations (4), (5), and (6), respectively.

(4) $$P=\frac{F_{z}V_{x}}{n\eta }$$

(5) $$T=\frac{FR}{ni\eta }$$

where *R* is the radius of the driving wheel.

(6) $$\;\;\;\;\;\;\;\;\;\;\;\;\;\;\;N=\frac{60V_{x}i}{\pi D}$$

The specific parameter requirements are calculated as shown in Table 2.

Under the premise of meeting the requirements of information parameters in Table 2, according to the driving ability required by the distance measurement accuracy of the sensor in Table 1, this paper chooses the Z55BLD300-36GU DC brushless motor.

**Figure 3:**
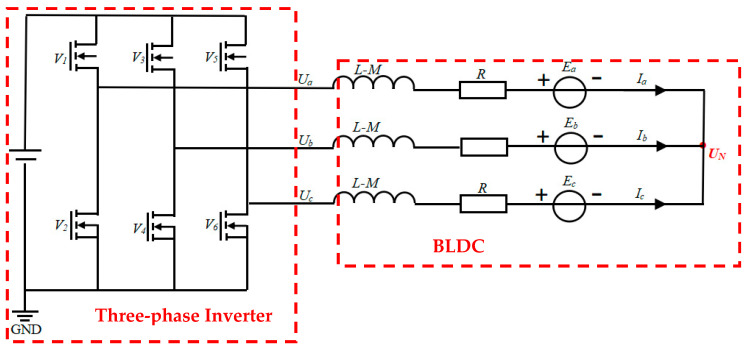
BLDC equivalent circuit.

For the realization of the motor control algorithm, according to the motor parameter characteristic hypothesis and model analysis of BLDC in reference [16], the simplified mathematical equation of the motor is established in this paper. The simplified equivalent circuit diagram of BLDC is shown in Figure 3. Through the analysis and prediction of the running state of the motor, different control principles can be designed, and the corresponding photoelectric sensor and sliding mode controller are used to obtain the speed feedback, respectively, so as to design the appropriate control strategy to achieve the desired speed regulation effect. The voltage equation of BLDC is as follows:

(7) $$\begin{array}{c} \left[\begin{array}{c} U_{a}\\ U_{b}\\ U_{c} \end{array}\right]=\left[\begin{array}{c} R\;\;\;0\;\;\;0\\ 0\;\;\;R\;\;\;0\\ 0\;\;\;0\;\;\;R \end{array}\right]\left[\begin{array}{c} I_{a}\\ I_{b}\\ I_{c} \end{array}\right]+B\frac{d}{dt}\left[\begin{array}{c} I_{a}\\ I_{b}\\ I_{c} \end{array}\right]+\left[\begin{array}{c} E_{a}\\ E_{b}\\ E_{c} \end{array}\right] \end{array}$$

where *R L* and *M* are phase resistance, phase inductance, and mutual inductance, respectively; *B* is the matrix $\left[\begin{array}{ccc} L-M\; & 0 & 0\\ 0 & L-M\; & 0\\ 0 & 0 & L-M\; \end{array}\right]$; *Ux Ix* and *Ex* are the three-phase voltage, phase current, and opposite electromotive force, respectively; *x* takes *a*, *b*, and *c* to represent the three-phase windings, respectively; *U_N_* is the star connected neutral voltage.

### 3.2. Speed Detection Circuit

In the low- and medium-speed-range BLDC control system, the motor output speed must be detected, and the measured speed is fed back to the PI controller to complete the speed closed-loop control. Considering the advantages of photoelectric sensors, such as their high accuracy and resolution, strong anti-interference ability, non-contact measurement, and high accuracy of displacement detection, the photoelectric encoder is selected as the BLDC output speed measuring device.

**Figure 4:**
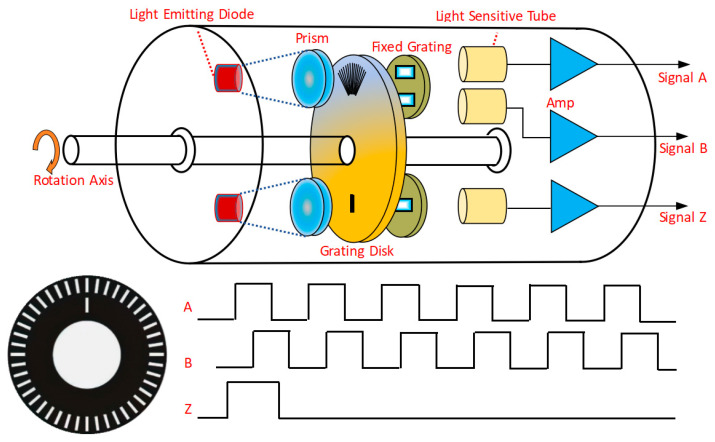
The structure schematic diagram of photoelectric encoder.

**Figure 5:**
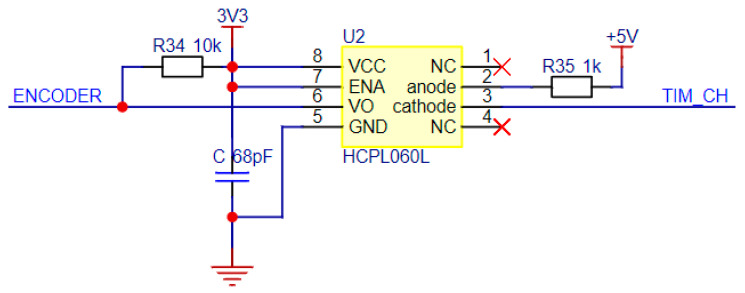
Photoelectric encoder interface circuit diagram.

The structure principle of the photoelectric encoder is shown in Figure 4, and the working principle is as follows:

1. The prisms convert the scattered light emitted by light-emitting diodes into parallel light, and fixed gratings ensure that this light enters the photosensitive tube. The grating disk rotates with the BLDC, and the photosensitive tube completes the optical–electrical signal conversion, and then outputs three sets of pulse square waves. They are AB two-phase square waves with a duty cycle of 50% and a phase difference of 90°, as well as Z-phase zero pulses. (One pulse is output per revolution).
2. Enter the single chip microcomputer through the interface circuit in Figure 5, then the single chip microcomputer captures the input pulse according to the interrupt service program, finally uses the frequency doubling technology (the rising edge and the falling edge of the AB two-phase square wave are both considered as pulses) to obtain a 4-fold frequency doubling pulse.
3. The BLDC speed is calculated using the periodic measurement method. This method obtains a high-frequency pulse with a known frequency and counts it. The calculation formula of the speed *n* is
  (8) $$n=\frac{1}{CT_{E}}=\frac{F_{0}}{CM_{1}}$$
  where *T_E_* is the counting time, which is determined by the interval time between two adjacent pulses of the encoder captured by the single-chip microcomputer; *M*_1_ is the corresponding number of counts; *C* is the total number of pulses in a single turn of the encoder; and *F*_0_ is the frequency of the pulses.

The interface circuit diagram of the photoelectric encoder is shown in Figure 5. HCPL060L is a photoelectric isolation device. The pulse signal generated by the photoelectric encoder enters the interface circuit and is then electrically isolated by HCPL060L. The isolated signal is processed after being captured by the timer of the single-chip microcomputer. This design can effectively prevent the influence of noise and interference in the encoder signal on STM32 and can improve the stability and reliability of the system.

When HCPL060L is in an extreme electromagnetic environment, its ability to resist unstable light interference worsens [17], which affects the accuracy of speed measurement and, ultimately, reduces the control accuracy and dynamic performance of the motor.

### 3.3. Vector Control Algorithm Rotation

BLDC is a complex system with multivariable, decoupling and nonlinear characteristics. The speed of BLDC cannot be effectively controlled within the required range by using ordinary inductive control (six-phase commutation method). Therefore, this paper adopts the FOC control algorithm.

The FOC algorithm, also known as Field-Oriented Control [18], is an advanced algorithm for AC motor control. The core idea is to transform the control problem of the AC motor into the control problem of the DC motor, so as to realize the efficient and accurate control of the AC motor.

**Figure 6:**
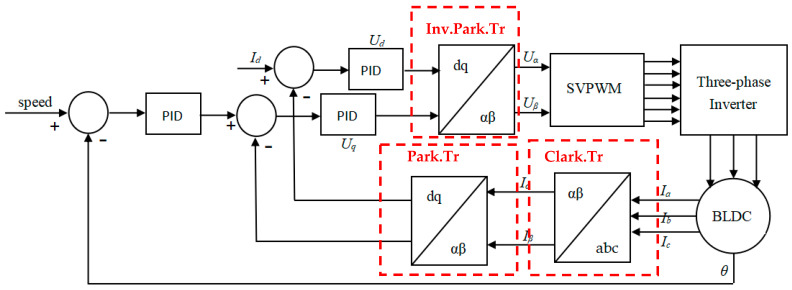
FOC control algorithm structure.

FOC aims to reduce the torque ripple and noise of the motor by accurately controlling the size and direction of the magnetic field, and can quickly respond to speed, rotor position, and torque commands, thereby achieving accurate control of torque and speed. As shown in Figure 6, the realization of the FOC algorithm is based on the Clark and Park coordinate transformation [18]. The FOC algorithm converts the three-phase stationary coordinate system (abc) into a two-phase rotating coordinate system (dq), which simplifies the control model and serves as the input to the PI controller. The Clark transform equation in the study is

(9) $$\begin{array}{c} \left[\begin{array}{c} I_{\alpha }\\ I_{\beta } \end{array}\right]=\sqrt{\frac{3}{2}}\;\left[\begin{array}{c} 1\;-\frac{1}{2}\;-\frac{1}{2}\\ 0\;\;\frac{\;\sqrt{3}}{\;2}\;-\frac{\sqrt{3}}{2}\; \end{array}\right]\left[\begin{array}{c} I_{a}\\ I_{b}\\ I_{c} \end{array}\right] \end{array}$$

where *αβ* is the two-phase stationary coordinate system. 

The Park transformation formula is

(10) $$\left[\begin{array}{c} I_{d}\\ I_{q} \end{array}\right]=\left[\begin{array}{c} cos\theta \;\;\;\;sin\theta \\ -sin\theta \;\;\;\;cos\theta \; \end{array}\right]\left[\begin{array}{c} I_{\alpha }\\ I_{\beta } \end{array}\right]$$

where *θ* is the current rotor position.

### 3.4. SVPWM Algorithm

The SVPWM algorithm [19] controls the switching state of the three-phase inverter and then generates a voltage or current output that is close to the ideal sinusoidal waveform. The SVPWM algorithm considers the inverter and the motor as a whole. By controlling the switching sequence of the inverter, the output voltage space vector achieves an ideal circular trajectory.

**Figure 7:**
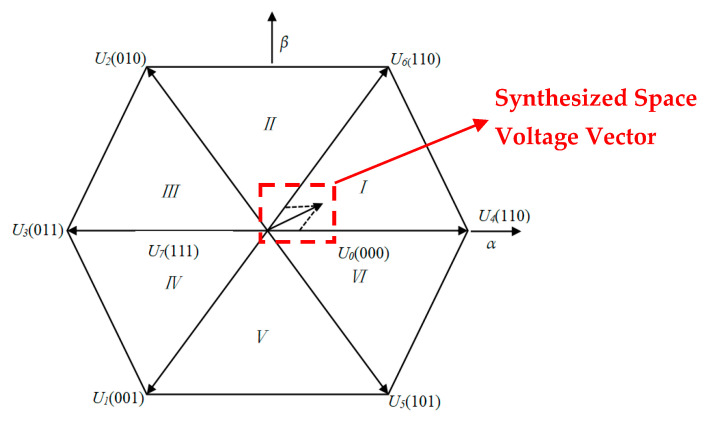
SVPWM space vector diagram.

The seven-segment SVPWM algorithm used in this paper and the generation method of the space voltage vector *U* in each sector are as follows: *U* consists of two zero vectors (*U*_0_, *U*_7_) and two adjacent non-zero vectors (*U*_1_~*U*_6_). The non-zero vector selected by the U in each sector of the synthesis corresponds to the state of the inverter MOS tube, as shown in Figure 7.

The generation of *U* first obtains each basic vector (*U*_0_~*U*_7_) through the switching state of the MOS tube and determines the sector where the current *U* is located according to the two adjacent non-zero vectors. Finally, according to the action time transformation of the above two pairs of adjacent vectors, the synthetic space voltage vector *U* in each direction is obtained, thereby driving the motor rotor to rotate smoothly.

The zero vector and the non-zero vector are combined to form the corresponding PWM waveform. Finally, the capture register (*CCR*) values corresponding to the three timers are obtained. According to the state combination of the power switch in the inverter circuit and the adjustment of its switching time, a space voltage vector with a circular trajectory is generated so that a uniform circular rotating magnetic field is generated in the AC motor.

In the vector control system, according to the selected control strategy, through the appropriate coordinate transformation, the components of the voltage space vector in the two-phase stationary coordinate system are obtained. Finally, through the SVPWM control, the specific algorithm steps are as follows:

1. According to the *U_α_* and *U_β_* obtained by the anti-Park transformation, the variable *U*_1_, *U*_2_, and *U*_3_ are defined. The calculation formulas of the three are as follows:

(11) $$\begin{array}{c} {U_{1}=U}_{\beta },\\ U_{2}=\frac{\sqrt{3}}{2}U_{\alpha }-\frac{1}{2}U_{\beta },\\ U_{3}=-\frac{\sqrt{3}}{2}U_{\alpha }-\frac{1}{2}U_{\beta }. \end{array}$$

2. The sector number of the space voltage vector (*I*–*VI* in Figure 7 ) is determined and calculated by reference voltage components (*U*_1_, *U*_2_, and *U*_3_).

(12) $$\begin{array}{c} N=sign\left(U_{1}\right)+2sign\left(U_{2}\right)+4sign\left(U_{3}\right) \end{array}$$

where the *sign* is the function, if *U_x_* > 0, the function value is 1; if *U_x_* < 0, the function value is 0.

And the corresponding sector is calculated based on Figure 7, sector location *N*.

3. Define the variables *X*, *Y*, and *Z*. According to the sector number obtained in Table 3, the action time of the corresponding basic space vector is determined.

(13) $$\begin{array}{c} X=\frac{\sqrt{3}T_{s}U_{\beta }}{U_{dc}}\\ Y=\frac{\sqrt{3}T_{s}}{U_{dc}}\left(\frac{\sqrt{3}}{2}U_{\alpha }+\frac{1}{2}U_{\beta }\right)\\ Z=\frac{\sqrt{3}T_{s}}{U_{dc}}\left(-\frac{\sqrt{3}}{2}U_{\alpha }+\frac{1}{2}U_{\beta }\right) \end{array}$$

where *T_s_* is the timer PWM period and *U_dc_* is the BLDC bus voltage.

According to the obtained *X*, *Y*, and *Z*, the values of the two non-zero vectors (rotated counterclockwise) corresponding to each sector are obtained.

When determining the output constraints for SVPWM modulation, in the process of software simulation, rounding is used in order to simplify the operation. Therefore, it is necessary to compare the working time *T_a_*, *T_b_* and the modulation period *T_s_*. If it does not meet the actual conditions, it needs to be processed.

(14) $$\begin{array}{c} T_{a}=\frac{T_{a}}{T_{a}+T_{b}}T_{s}\\ T_{b}=\frac{T_{b}}{T_{a}+T_{b}}T_{s} \end{array}$$

4. The PWM generation of a single-chip microcomputer adopts the center alignment mode. In this mode, the single-chip microcomputer can generate the symmetrical PWM waveform, and an accurate synthetic magnetic field vector can be generated by the SVPWM algorithm. Therefore, the setting of the *CCR* value in the timer should be determined first.

First, the following three variables are defined: *T*_1_, *T*_2_, and *T*_3_. According to the values of *T_a_* and *T_b_* in each sector, as shown in Table 4, these three variables can be obtained.

(15) $$\begin{array}{c} T_{1}=\frac{{(T}_{s}-T_{a}-T_{b})}{4}\;\\ T_{2}=T_{1}+\frac{T_{a}}{2}\\ T_{3}=T_{2}+\frac{T_{b}}{2} \end{array}$$

The corresponding relationship between the three-phase voltage switching points *CCR*_1_, *CCR*_2_, and *CCR*_3_ and each sector is shown in Table 5.

5. On the basis of the principle of the minimum number of MOS tube-switching operations, the order and time of each sector voltage vector are determined. Finally, the value of CCR in the timer setting of the single chip microcomputer is determined according to Table 5.

**Figure 8:**
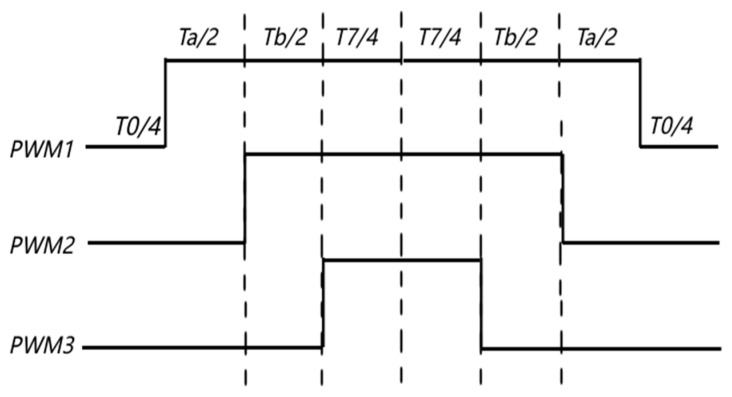
Three-phase PWM waveform of the first sector.

Taking the first sector as an example, the three-phase modulation wave generated by it is shown in Figure 8 in the period *Ts*. The order of appearance of the voltage vectors in the figure is 0→4→6→7→7→6→4→0.

In summary, the SVPWM algorithm process includes the sector judgment of the reference voltage vector, the calculation of the non-zero vector and the zero vector action time of each sector, and the determination of the switching point of each sector vector. Then, comparing the corresponding frequency triangular carrier signal with each sector vector switching point, the six PWM pulse signals required by the three-phase inverter are finally generated.

### 3.5. Sliding Mode Observer

For the output-speed feedback of the photoelectric encoder under the vector control algorithm, it is not sufficient to meet the AGV speed requirements. In order to improve the response speed of the drive system and improve the accuracy of the position feedback system, on the basis of the vector control strategy, the sliding mode observer is used in the high-speed stage of BLDC to strengthen the control of the output speed of the motor.

SMO is an advanced algorithm [20] for sensorless vector control of motors. The algorithm is mainly used to estimate the rotor position and speed of the motor in real time, so as to realize the accurate control of the motor. SMO is an observer based on the theory of sliding mode control. The core concept of the SMO is to design a sliding surface so that the system state can slide along the sliding surface after reaching the sliding surface, and finally realize the observation of the position and speed of the rotor.

1. The first step is to design the SMO and select a suitable control function to reduce the output chattering problem. The mathematical model of the SMO is established based on the motor voltage equation in the stationary coordinate system, and the voltage equation in the stationary coordinate system obtained by Clark transformation of (7) is

(16) $$\left[\begin{array}{c} U_{\alpha }\\ U_{\beta } \end{array}\right]=\left[\begin{array}{c} R+pL_{d}\;\;\;\;\;\;\;\;0\\ 0\;\;\;\;\;\;\;\;R+pL_{q} \end{array}\right]\left[\begin{array}{c} I_{\alpha }\\ I_{\beta } \end{array}\right]+\left[\begin{array}{c} E_{\alpha }\\ E_{\beta } \end{array}\right]$$

The counter electromotive force contains the motor speed information, and the voltage equation becomes the current equation in the same coordinate system as follows:

(17) $$\begin{array}{c} \left[\begin{array}{c} \frac{d}{dt}I_{\alpha }\\ \frac{d}{dt}I_{\beta } \end{array}\right]=\left[\begin{array}{cc} -\frac{R}{L_{d}} & 0\\ 0 & -\frac{R}{L_{d}} \end{array}\right]\left[\begin{array}{c} I_{\alpha }\\ I_{\beta } \end{array}\right]+\frac{1}{L_{d}}\left[\begin{array}{c} U_{\alpha }\\ U_{\beta } \end{array}\right]-\frac{1}{L_{d}}\left[\begin{array}{c} E_{\alpha }\\ E_{\beta } \end{array}\right] \end{array}$$

2. To obtain an estimate of the counter electromotive force, a sliding mode observer is designed according to the observation current equation in the BLDC stationary coordinate system:

(18) $$\begin{array}{c} \left[\begin{array}{c} \frac{d}{dt}\hat{I_{\alpha }}\\ \frac{d}{dt}\hat{I_{\beta }} \end{array}\right]=A\left[\begin{array}{c} \hat{I_{\alpha }}\\ \hat{I_{\beta }} \end{array}\right]+\frac{1}{L_{d}}\left[\begin{array}{c} U_{\alpha }\\ U_{\beta } \end{array}\right]-\frac{1}{L_{d}}\left[\begin{array}{c} v_{\alpha }\\ v_{\beta } \end{array}\right] \end{array}$$

where $\hat{I_{\alpha }}$ $\hat{I_{\beta }}$ are the current observations in the stationary coordinate system; *v_α_ v_β_* are the control inputs to the observer, which can be expressed as follows:

(19) $$\begin{array}{c} \left[\begin{array}{c} v_{\alpha }\\ v_{\beta } \end{array}\right]=\left[\begin{array}{c} k\cdot sgn\left(\hat{I_{\alpha }}-I_{\alpha }\right)\\ k\cdot sgn\left(\hat{I_{\beta }}-I_{\beta }\right) \end{array}\right] \end{array}$$

where *k* is the slip film gain and *sgn* is the sign function [21].

The appropriate *k* value can make the observation current converge to the actual current more quickly and can reduce the jitter of the observation current and the SMO output speed after the stability, so as to improve the speed extraction accuracy.

3. In order to better reduce the chattering and remove other high-frequency noise in the system, a low-pass filter is added after the output *v_α_ v_β_* of the sliding mode observer, and its mathematical model in the frequency domain is

(20) $$\begin{array}{c} H\left(j\omega \right)=\frac{\omega_{c}}{\omega_{c}+j\omega_{e}} \end{array}$$

where $\omega_{c}$ is the cutoff frequency and $\omega_{e}$ is the is the identification speed.

4. The rotor position and speed are estimated by phase-locked loop (PLL). The PLL [22] algorithm is widely used in digital signal processing, especially in the fields of communication, power electronics, and motor control. Its main purpose is to track the input signal’s phase to extract its frequency and phase information. The equation for the PLL estimation of rotor speed is as follows:

(21) $$\begin{array}{c} \hat{\omega }=\left(-\hat{E_{\alpha }}cos\hat{\theta }-\hat{E_{\beta }}sin\hat{\theta }\right)\left(k_{p}+\frac{k_{i}}{s}\right) \end{array}$$

where $\hat{E_{\alpha }}$ $\hat{E_{\beta }}$ is the observed value of the back electromotive force after the filter; $k_{p}$ is the proportional value of the PI controller, and $k_{i}$ is the integral value of the PI controller.

The estimated speed of the rotor extracted by the phase-locked loop needs to be output by the integrator, which is the rotor position estimation.

## 4. Simulation of Drive and Control System

### 4.1. Design of Low- and Medium-Speed-Range Control Systems

**Figure 9:**
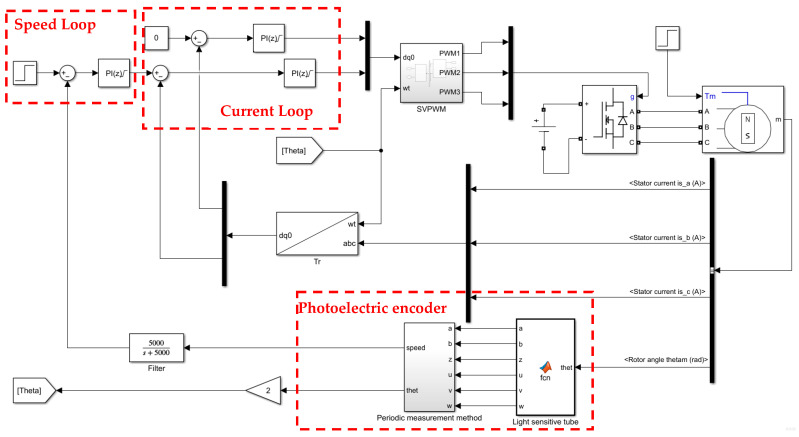
Dual closed-loop control system simulation.

At low and medium speeds, the photoelectric encoder is used for dual closed-loop vector control, and the PI controller is used in the speed outer loop and the current inner loop. The rotation axis of the photoelectric encoder follows the rotation of the motor, and the mechanical angle of the motor output rotor is used as the input of the photoelectric encoder. Figure 4 shows that the photosensitive tube generates an ABZ three-phase pulse, and the current motor speed is calculated through Formula (8). And the output speed of the photoelectric encoder is passed through the low-pass filter, so that the high-frequency noise can be removed and the output speed chattering can be reduced and used as the feedback of the PI controller. The cut-off frequency of the low-pass filter is the output PWM frequency of the SVPWM algorithm. The simulation model of the system is shown in Figure 9.

### 4.2. PI Parameters Tuning

The use of the critical ratio method, attenuation curve method, and other engineering rectification methods cannot adjust the PI parameters in time according to the external environment, which will affect the system’s regular operation. In this paper, the theoretical calculation setting method [23] is selected, so the calculation amount is reduced by the Laplace transform. The premise of Laplace transformation is a linear constant system, so the transfer function is constructed under the assumption of ignoring the coupling term in the current loop controller.

1. Calculate the transfer function of the BLDC in the dq axis coordinate system according to Equation (7).

(22) $$\begin{array}{c} u_{d}=Ri_{d}+L_{d}\frac{d_{id}}{d_{t}}\\ u_{q}=Ri_{q}+L_{q}\frac{d_{iq}}{d_{t}} \end{array}$$

After the Laplace transform, the transfer function is

(23) $$\begin{array}{c} G_{d\left(s\right)}=\frac{i_{d\left(s\right)}}{u_{d\left(s\right)}}=\frac{1}{R+L_{d}s}\\ G_{q\left(s\right)}=\frac{i_{q\left(s\right)}}{u_{q\left(s\right)}}=\frac{1}{R+L_{q}s} \end{array}$$

**Figure 10:**
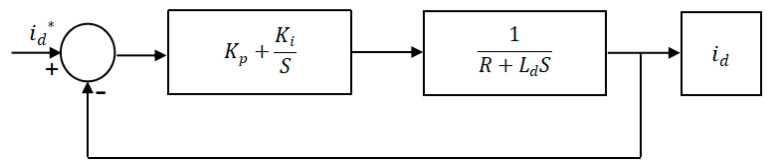
Current closed-loop transfer function.

2. Using the parallel PI controller, under the premise of ignoring the system delay link and filtering the feedback current, the open-loop transfer function of the motor current loop system shown in Figure 10 is

(24) $$\begin{array}{c} G_{open\left(s\right)}=\frac{i_{d\left(s\right)}}{{i_{d\left(s\right)}}^{*}}=\left(k_{p}+\frac{k_{i}}{s}\right)\left(\frac{1}{R+L_{d}s}\right) \end{array}$$

Simplify the current loop closed-loop transfer function to obtain

(25) $$G_{close\left(s\right)}=\frac{k_{p}s+k_{i}}{L_{d}s^{2}+\left(k_{p}+R\right)s}$$

Then create the following equation

(26) $$\begin{array}{c} \begin{array}{c} k_{p}=L_{d}\omega_{c}\\ k_{i}=R\omega_{c} \end{array} \end{array}$$

where $\omega_{c}$ is the current loop bandwidth, and related to the time constant τ of the motor, which can be substituted to simplify the closed-loop transfer function

(27) $$\begin{array}{c} G_{close\left(s\right)}=\frac{\omega_{c}}{s+\omega_{c}} \end{array}$$

This is calculated using the following formula:

(28) $$\begin{array}{c} \tau =min\left[\frac{L_{d}}{R},\frac{L_{q}}{R}\right]\\ \omega_{c}=\frac{2\pi }{\tau } \end{array}$$

The poles of the open-loop transfer function are negative, so the $k_{p}$ $k_{i}$ of the current loop can be obtained on the premise that the transfer function is stable.

3. The parameters of the speed-loop PI regulator are set by the following formula

(29) $$\begin{array}{c} K_{p}=\frac{\beta J}{1.5p_{n}\psi_{f}}\\ K_{i}=\beta K_{p}\;\;\;\;\;\;\;\; \end{array}$$

where *β* is the expected bandwidth of the speed ring, which is taken as 50; *J* is the moment of inertia, *P_n_* is the number of pole pairs; $\psi_{f}$ is the motor flux linkage.

4. PI parameters under dual closed-loop vector control can be obtained.

Substituting the Table 6 parameters into the PI controller in Figure 9.

5. For the coupling term shown above, through comparison in the simulation experiment, it can be seen that the coupling term is used as the error input of the current loop PI controller, and the current waveform output by the motor is better.

### 4.3. Construction of High-Speed Range Control System

**Figure 11:**
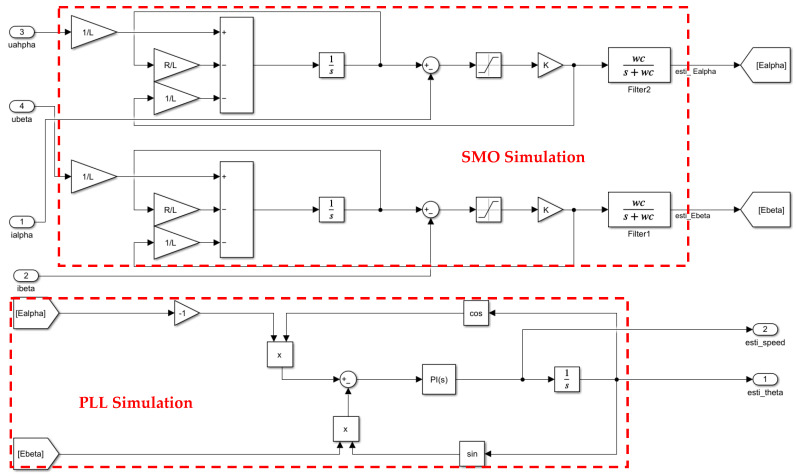
SMO simulation module.

In order to obtain a better speed output response curve, SMO is used to estimate the speed and position of the motor rotor at high speed, and the estimated value is used to replace the speed and position value detected by the photoelectric encoder in the original system. The sliding mode observer is a nonlinear observer. Compared with other state observers, it has excellent robustness based on high dynamic response capability [24]. SMO uses the sign function to replace the actual value of the deviation, which not only effectively copes with external interference, but also effectively improves the stability of the control system. Figure 11 shows the SMO simulation module [25].

The high-speed stage simulation build process is as follows:

1. Firstly, for the BLDC output three-phase current and voltage, the two-phase stationary coordinate system (*αβ*) is obtained by anti-Park transformation, and the differential of the actual value of the current is obtained by substituting this into Formula (17). Then, the actual value of the current in the two-phase stationary coordinate system can be obtained by integrating the differential value. Finally, according to Formula (18), the differential of the corresponding observation value is obtained.

2. To keep the observer state in the sliding mode plane $\hat{I_{\alpha }}-I_{\alpha }$ all the time, according to the sliding mode control law of Equation (19), the estimated value of the back electromotive force can be obtained, and the estimated value of the back electromotive force is filtered as the PLL input.

3. The PLL simulation module is built according to (21), and the output value is the rotor speed observation value. After the speed is integrated, it is the rotor position observation value.

The method of calculating each parameter in the simulation module is the experimental debugging method. Synovial gain *K* value is 200; the value of *wc* in the filter is 4000; in the PI controller of PLL, *Kp* = 43, *Ki* = 20,000.

## 5. Simulation Analysis

To verify the validity and reasonableness of the proposed method, according to the simulation model of Matlab/Simulink (R2022a), the parameters of the BLDC are set in the full speed range as follows: line resistance *R* = 0.4 Ω, line inductance *Ld* = *Lq* = 0.8 mH, moment of inertia *J* = 0.000021 kg·m^2^, flux linkage *Ψ* = 0.04457 Wb, and number of pole pairs *p* = 2.

**Figure 12:**
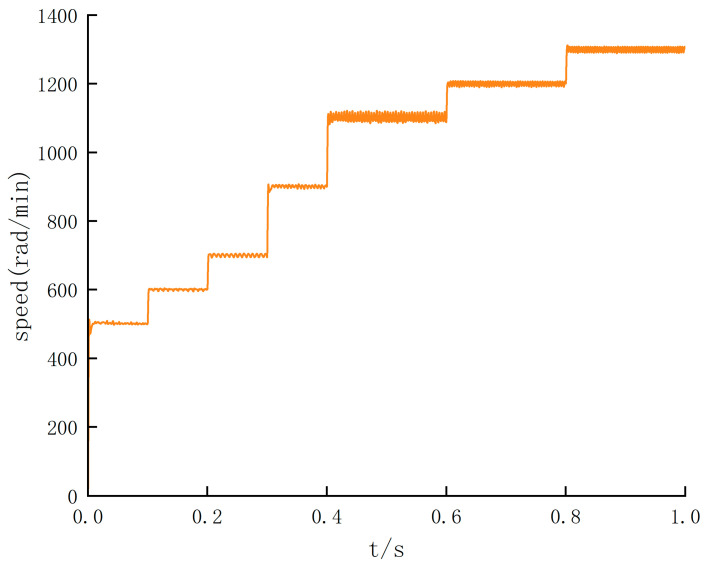
Speed feedback waveform of photoelectric encoder.

In order to study the speed defect of photoelectric encoder detection, the photoelectric encoder is used to measure the speed. Under the premise of not exceeding the maximum speed set in Table 2, under the premise of not exceeding the maximum speed set in Table 2, BLDC operates at variable speed within the speed stages of 500, 600, 700, 900, 1100, 1200, and 1300 rad/min, respectively. The simulation results are shown in Figure 12.

**Figure 13:**
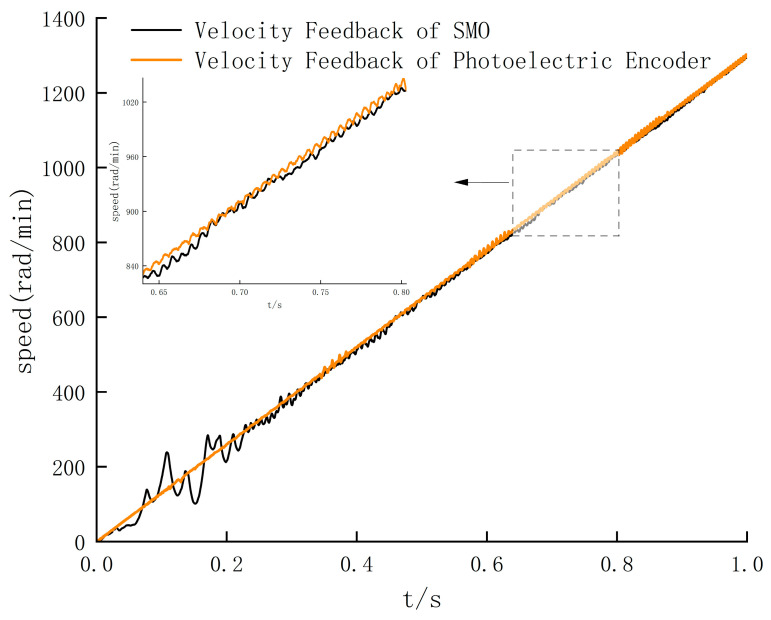
Comparison of full-speed-range velocity waveform.

The simulation results show that the output speed waveform of the motor is relatively stable in the low-speed operation stage. Still, there is an apparent jitter in the speed output waveform at high speed. Therefore, based on this defect, the SMO algorithm is added for speed feedback. In order to determine the switching threshold of the SMO algorithm, the BLDC uniform acceleration driving experiments can be conducted, and the simulation is carried out on the basis of two different algorithms. The simulation results are shown in Figure 13.

The simulation results in Figure 12 and Figure 13 show that there is no overshoot at the time of speed switching, and the robustness of the system is verified. And it can be explained that under the premise of the feedback speed of the photoelectric encoder, the AGV speed output is stable when driving at low speed. Figure 13 shows that when the BLDC speed exceeds 900 rad/min, the output waveform of the speed feedback of the encoder is seriously jittered. Still, on the basis of the SMO algorithm, the output waveform of the speed feedback is stable.

In order to accurately determine the threshold of SMO algorithm switching, this paper selects the initial speed of 890 rad/min, lets the speed increase in turn, and compares the feedback speed waveforms of the two control methods.

In order to further reflect the chattering of the velocity waveform and obtain the switching threshold more accurately, Table 7 shows the standard deviation of each velocity stage obtained by Bessel formula.

**Figure 14:**
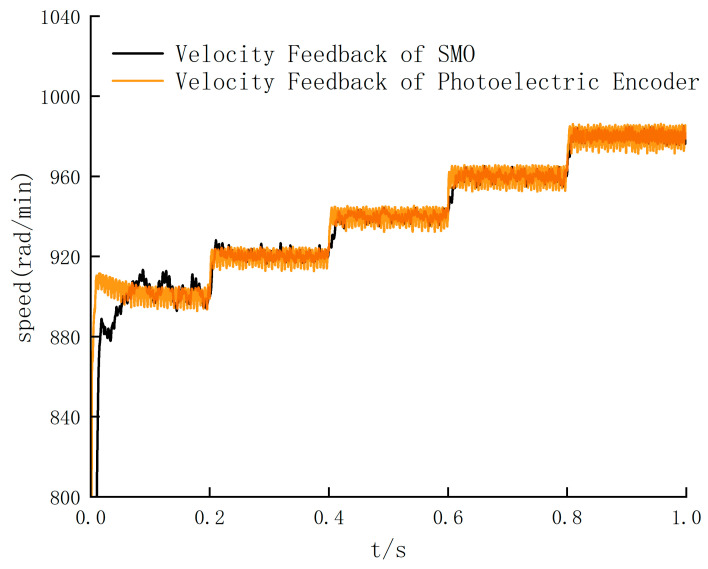
Feedback speed waveform comparison.

**Figure 15:**
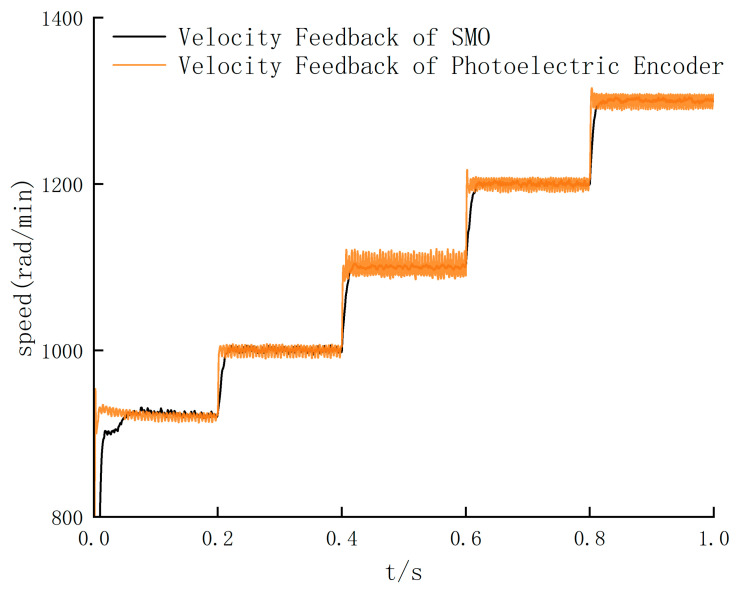
Comparison of speed feedback waveforms in high-speed range.

According to Figure 13, the threshold of the SMO algorithm switching is around 900 rad/min. According to the standard deviation values of Figure 14 and Table 7, the standard deviation of the photoelectric encoder speed feedback is larger than that of the SMO algorithm after the speed exceeds 920 rad/min, so the threshold is 920 rad/min. In order to further verify the effectiveness of the SMO algorithm in the high-speed range, the sliding mode observer is switched to the simulation control in the high-speed stage. Five high-speed ranges with noticeable differences and significant jitter are selected, respectively, and then the speed feedback waveforms of the SMO algorithm and photoelectric encoder are compared. The results are shown in Figure 15.

The simulation results in Figure 13 and Figure 15 show that the output speed waveform jitter phenomenon is significantly optimized in the high-speed range. Combined with the comparison results of 920–1300 rad/min range, the maximum chattering value can be reduced from 10.8 to 5.2 rad/min after the speed reaches a steady state. It can be concluded that the speed feedback value is more concentrated, closer to the real value, and more in line with the control requirements. Therefore, the output error is significantly reduced, and the system stability is improved, which is in line with the expected goal.

**Figure 16:**
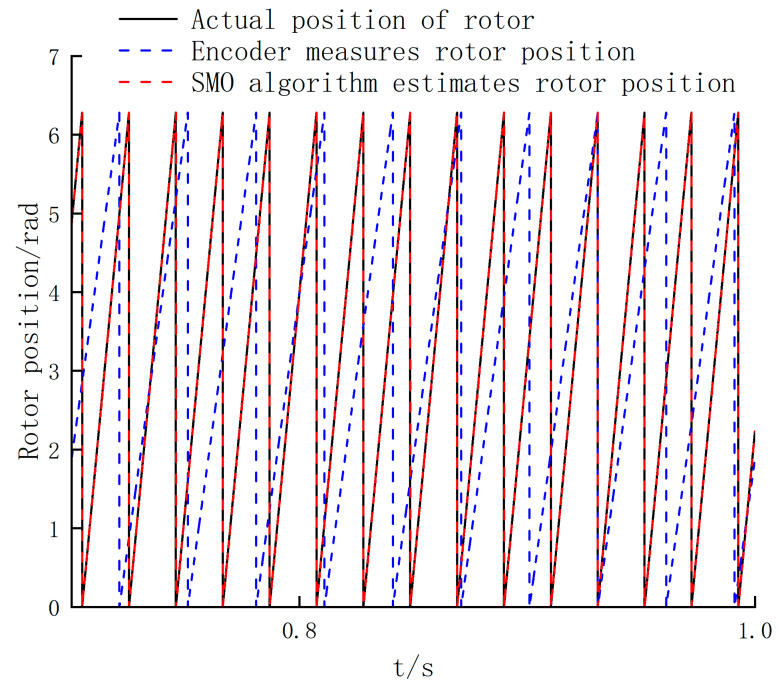
Comparison of rotor position waveforms under three different conditions.

In vector control, the phase error is more important than the velocity error. The phase error is the radian difference between the actual rotor position and the rotor position measured by the photoelectric encoder, and also the radian difference between the actual rotor position and the rotor position estimated by SMO. In order to study the superiority of the SMO algorithm in the high-speed range, the rotor positions of the three cases are compared in the high-speed range of BLDC. The phase error is shown in Figure 16.

According to the observation angle of the motor in the high-speed range in Figure 16, it can be seen that there is a deviation between the rotor phase measured by the photoelectric encoder and the actual value, which will cause an increase in chattering and a decrease in control accuracy. However, there is no phase error under the SMO algorithm, which verifies the feasibility [26] of the SMO control strategy within the high-speed range.

**Figure 17:**
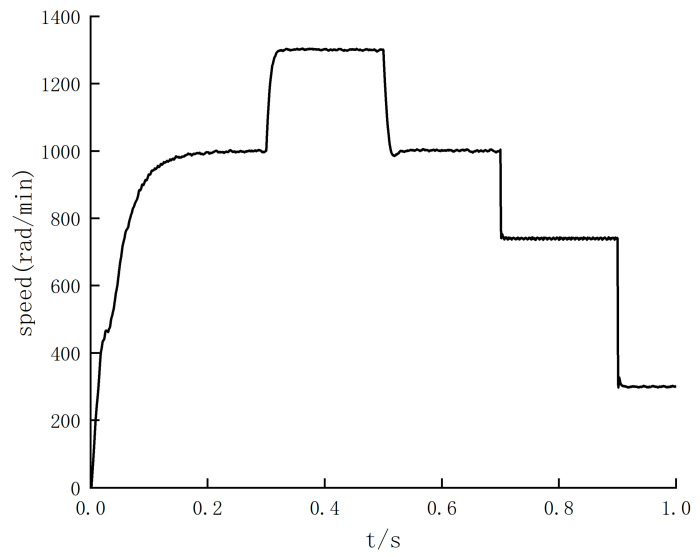
Full-speed-range load constant deceleration feedback waveform.

Because the actual operation mode of AGV in the intelligent garage is more complex, in order to verify the performance of the full-speed-range hybrid control strategy in the comprehensive case, this paper carries out variable speed operation of AGV in the full-speed range under the premise of BLDC load 0.3 N·m. By sorting out the data, the simulation results of five stages with obvious jitter are obtained, as shown in Figure 17.

The simulation results show that the BLDC output speed waveform is stable under the hybrid control strategy. Although there is an overshoot, it does not exceed 1.5%, and the maximum chattering does not exceed 6.1 rad/min. It can run stably with or without load conditions. The control algorithm satisfies the instantaneous acceleration and deceleration of BLDC. Based on the high-precision measurement distance of obstacles in the garage, this paper studies the control of BLDC and considers that the specific position of AGV is the integration of speed in the running time, so the specific position of AGV in the intelligent garage can be accurately determined, so as to avoid collision and realize the high safety of AGV in the garage. Therefore, the research content of this paper meets the requirements of AGV driving reliability and safety.

## 6. Conclusions

This paper takes the design of an intelligent garage AGV obstacle avoidance system as the object. Under the premise that the accuracy of the ranging module is 0.17 cm, the design of the driving system in the full speed range is discussed, and the simulation model is built in Matlab/Simulink. According to the BLDC speed output curve under this system, it is found that the dual closed-loop vector control based on a photoelectric sensor has an excellent control effect in the low- and medium-speed stages, but the control effect does not meet the actual needs in the high-speed stage, so the speed measurement is switched to SMO to improve the stability of the speed output curve. This method can not only reduce the jitter value by 50%, but can also remove the phase error of encoder speed measurement. In the high-speed stage of BLDC, compared with the sensing FOC motor control, the sensorless sliding mode observation algorithm directly adjusts the system gain according to the motor running state, which has strong robustness. Two different speed sensors are used in the full speed range, a reasonable sensor switching threshold is selected, and the output speed curve is better in terms of dynamic and steady-state performance, which aligns with the actual production needs.

Through the output curve under the SMO, it is known that the speed waveform still fluctuates after reaching stability, which is related to the following parameters: the parameters of the motor, the synovial gain in the SMO algorithm, the parameters of the low-pass filter, and the PI controller parameters in the phase-locked loop. Subsequent research will optimize these parameters. Finally, it should be considered that the over-modulation of the PI controller during the acceleration of the motor will cause jitters in the feedback speed. In addition, considering that during the acceleration of the BLDC motor, the over-modulation of the PI controller will also cause the jitter of the feedback speed. Therefore, when the chattering gap between the output speed of the photoelectric encoder and the SMO is slight, the modulation effect of the PI controller has a great influence, and the superiority of the SMO is difficult to reflect. Finally, this will be further studied in the future.

## Figures and Tables

**Table 1:** Data processing results.

	Maximum Error/cm	Minimum Error/cm	Average Error/cm
Raw data	0.75	0	0.28
Filtered data	0.58	0.004	0.17

**Table 2:** Drive motor parameter requirements.

Rating W	Rated Torque N·m	Rated Speed r/min
121.4	0.96	1230

**Table 3:** The corresponding relationship between N and the sector.

N	3	1	5	4	6	2
Sector	1	2	3	4	5	6

**Table 4:** Vector action time for each sector.

N	1	2	3	4	5	6
T_a_	Z	Y	−Z	−X	X	−Y
T_b_	Y	−X	X	Z	−Y	−Z
T_0_(T_7_)	(T_s_ − T_a_ − T_b_)/2					

**Table 5:** Time-switching points of each sector.

N	1	2	3	4	5	6
CCR_1_	T_2_	T_1_	T_1_	T_3_	T_3_	T_2_
CCR_2_	T_1_	T_3_	T_2_	T_2_	T_1_	T_3_
CCR_3_	T_3_	T_2_	T_3_	T_1_	T_2_	T_1_

**Table 6:** PI control parameters.

	K_p_	K_i_
Current ring	2.51	1256.6
RPM ring	0.01	0.5

**Table 7:** Comparison of standard deviation of two algorithms in each speed stage.

Speed (rad/min)	Standard Deviation—Encoder	Standard Deviation—SMO
890	9.93	13.70
900	5.76	12.66
910	2.00	4.03
920	3.14	2.12
930	2.15	1.99
940	3.27	2.00
950	2.41	1.84
960	3.67	2.22
970	2.48	2.44
980	4.03	2.14

## Data Availability

All data are contained within the article.
